# Role of LINC01133 in Osteogenic Differentiation of Dental Pulp Stem Cells by Targeting miR-199b-5p

**DOI:** 10.3290/j.ohpd.b2960495

**Published:** 2022-04-27

**Authors:** Qiaorui Shi, Ming Zheng

**Affiliations:** a Physician, Department of Special Clinic, School and Hospital of Stomatology, Fujian Medical University, Fujian Stomatological Hospital, Fuzhou, Fujian, P.R. China. Idea, experimental design, performed the experiments, analysed the data, wrote the manuscript, read and approved the final version for publication.; b Chief Physician, Department of Oral Prosthetics, School and Hospital of Stomatology, Fujian Medical University, Fujian Stomatological Hospital, Fuzhou, Fujian, P.R. China; Fujian Key Laboratory of Oral Diseases, Laboratory of Oral Tissue Engineering, Fujian Medical University, Fuzhou, Fujian, P.R. China. Idea, experimental design, provided crucial materials, supervised the study, read and approved the final version of the manuscript for publication.

**Keywords:** AKT3, DPSCs, competitive endogenous RNA, LINC01133, miR-199b-5p, osteogenic differentiation

## Abstract

**Purpose:**

Recently, increasing attention has been paid to the function of long non-coding RNAs (lncRNAs) in osteogenic differentiation (OD) of dental pulp stem cells (DPSCs). LINC01133 was reported to have a close relationship with tumorigenesis for multiple cancers, but no study has yet explored the role of LINC01133 in modulating OD of DPSCs.

**Materials and Methods:**

Alizarin red S (ARS) staining and alkaline phosphatase (ALP) staining were perfomed to assess the OD potential of DPSCs. Osteogenic markers including runt-related transcription factor 2 (RUNX2), osterix (OSX) and ALP expression levels in DPSCs were monitored by qRT-PCR and Western blot before and after cell transfection. Luciferase reporter gene assay detected the relationship between LINC01133 and miR-199b-5p.

**Results:**

The expression of LINC01133 was low, while miR-199b-5p was increasingly expressed during OD of DPSCs. Overexpression of LINC01133 in DPSCs resulted in decreased expression of RUNX2, OSX, ALP, DSPP and DMP1, whose expression was reversed in DPSCs after transfections of miR-199b-5p overexpression. Co-transfection of pcDNA3.1-LINC01133 and miR-199b-5p mimic led to elevated expression of RUNX2, OSX, ALP, DSPP and DMP1 compared with pcDNA3.1-LINC01133 transfection alone. LINC01133 served as a sponge of miR-199b-5p. AKT3 was verified as a downstream effector of miR-199b-5p in DPSCs.

**Conclusion:**

LINC01133 inhibits the OD of DPSCs by upregulating AKT3 via sponging miR-199b-5p, which may act as a potential diagnostic biomarker for dentin regeneration in the dental pulp.

**Supplementary Figure:**
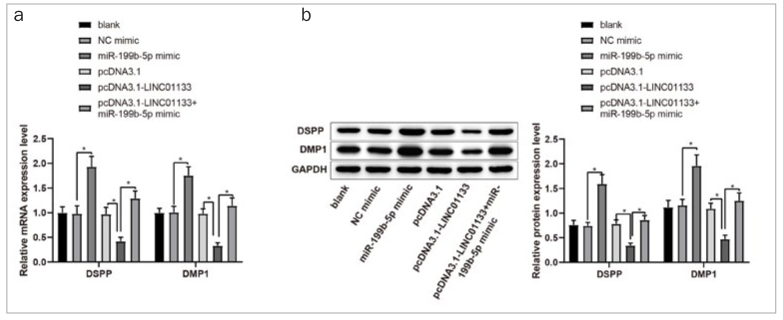
The expressions of DSPP and DMP1 in DPSCs were determined by qRT-PCR and Western Blot. After transfection with pcDNA3.1-LINC01133 or/and miR-199b-5p mimic, the mRNA and protein levels of DSPP and DMP1 were measured. DPSCs, dental pulp stem cells; DSPP, dentin sialophosphoprotein; DMP1, dentin matrix protein1.

Pulp tissue, located in the pulp cavity, is the only soft tissue in tooth and is of essential importance in tooth nutrition, innervation, and immunocompetency.^2,11^ Pulpitis is a chronic or acute inflammation of dental pulp, which can be caused by trauma or caries^4^ and may consequently lead to loss of regeneration ability of dental pulp cells, making the complete healing of pulpitis difficult.^31^ Root canal treatment is one of the most frequent treatment approaches for pulpitis,^17,18^ but it can only remove the diseased pulp and will eventually lead to tooth brittleness.^23^ Hence, exploration of alternative treatment options for treating pulpitis is of great importance.

Among mesenchymal stem cells (MSCs), dental pulp stem cells (DPSCs) are adherent clonogenic cells with highly proliferative ability and multidirectional differentiation ability into chondrocytes, adipocytes, neural cells and osteoblasts.^5,26^ These properties make DPSCs an attractive choice not only for dental diseases but also for systemic diseases.^7^ Nowadays, the therapeutic potential of DPSCs has been identified in tooth repair for its ability for osteogenic differentiation (OD).^22^ However, the molecular mechanisms regulating OD of DPSCs warrant further investigation.

Long non-coding RNAs (lncRNAs) are a large class of RNAs with a length of more than 200 nucleotides, and lack translation ability.^8,10^ LncRNAs have been shown to be regulatory factors for OD of MSCs^15,41^ by directly or indirectly mediating the osteogenesis process. In recent decades, altered expression of a variety of lncRNAs and small non-coding microRNAs (miRNAs) has been observed during OD of multiple stem cells, including DPSCs.^30,34^ For instance, lncRNA ANCR suppressed OD via negatively regulating miR-758.^24^ LncRNA LEF1-AS1 was also found to function as a promoter of miR-24-3p during OD of DPSCs.^34^ These observations indicated the key roles of lncRNAs and miRNAs in regulating the differentiation of DPSCs. LINC01133 was identified as an emerging tumor-associated lncRNA in cervical cancer,^6,39^ pancreatic cancer^40^ and osteosarcoma.^19^ Increasing LIN01133 (also termed lncRNA PAGBC) was also reported to induce OD of adipose-derived MSCs^29^ as well as human bone narrow MSCs.^27^ Nevertheless, how LIN01133 regulates OD in MSCs remains to be further explored, and currently no research has been done on the regulation of LIN01133 in OD of DPSCs. Among all the miRNAs, miR-199b-5p as a regulator to promote OD of human bone marrow stromal cells has been reported.^42^ Furthermore, miR-199b-5p was also found to inhibit OD in ligamentum flavum cells,^28^ suggesting the possibility of miR-199b-5p in regulating OD in stem cells. Online software showed the possible binding sites between LIN01133 and miR-199b-5p; therefore, we speculate that LIN01133 and miR-199b-5p may have certain role to play during OD of DPSCs.

In this work, we elucidated the novel function of LINC01133 in regulation of osteogenesis via sponging miR-199b-5p to regulate AKT3 expression. These findings showed the significance of LINC01133/miR-199b-5p/AKT3 axis in OD of DPSCs and thus further provided a potent therapeutic approach for tooth regeneration.

## Materials and Methods

### Isolation and Culture of DPSCs

Dental pulp tissue was extracted from third molars of healthy adult subjects in the Department of Stomatology of Fujian Stomatological Hospital. The harvesting of clinical tissues was approved by the ethics committee of Fujian Stomatological Hospital. Written informed consent was obtained from all donors. Immediately after the teeth were extracted, the pulp tissue was incubated in a pre-cooled (4°C) Dulbecco’s Modified Eagles Medium (DMEM) containing double antibiotics. The subsequent operations were carried out on an ultraclean bench. The teeth were rinsed repeatedly with PBS containing penicillin and streptomycin (100 U/ml). Then the pulp tissue of permanent and deciduous teeth were harvested aseptically and rinsed repeatedly with PBS, after which the tissues were dissected into small pieces with dimensions of 1 mm x 1 mm x 1 mm. Human DPSCs were obtained as described in previous reports.^16,25^ For extraction of DPSCs, the dental pulp tissues were digested in a solution of 3 mg/ml collagenase type I and 4 mg/ml dispase for 1 h at 37°C, with water bath shaking for complete digestion. Thereafter, the tissues were centrifuged at 1000 rpm/min for 10 min, with the supernatant being removed. Then, the culture medium containing 20% fetal bovine serum (FBS, Thermo Fisher Scientific; Waltham, MA, USA) was applied to resuspend the cells, and the cell suspension was filtered with a 100-μm steel mesh. After cell counting, the cell concentration was adjusted and the cells were inoculated into 96-well plates (1–2 x 10^3^ per well) for 7–14 days of cell culture. When cell colonies (≥ 50 cells) were observed, the cells were subjected to cell expansion and inoculated into 6-well plates (5 x 10^4^ cells/ml) before incubation with 10% FBS-supplemented DMEM. After cell culture, cells with 70% confluence were incubated with OD solution (containing 50 ug/ml vitamin C, 10% FBS contained α-MEM, 1 x 10^-8^ mol/l dexamethasone, 5 mmol/Lβ- sodium glycerophosphate; Cyagen Biosciences; Guangzhou, China) for continuous culture. The solution was changed every 3 days and cells were observed daily under a microscope.

### Alizarin Red S (ARS) Staining

After 21 days of osteogenic induction, cells were washed 2 or 3 times with PBS. Then, the PBS solution was removed and 4% paraformaldehyde was added for 15-min cell fixation at room temperature. After that, the cells were rinsed with double distilled water (ddH_2_O) and stained with ARS solution (Beyotime Biotechnology; Shanghai, China) at room temperature. After 30 min of ARS staining, the cells were rinsed with repeatedly ddH_2_O. The cells were observed and photographed by a microscope (IX50, Olympus; Tokyo, Japan).

### Alkaline Phosphatase (ALP) Staining

After 21 days of osteogenic induction, cells were washed 2 or 3 times with PBS. After PBS was removed, the cells were fixed with 2 ml of 4% paraformaldehyde for 15 min at room temperature, followed by ddH_2_O washing. ALP solution (Mao Kang Biotechnology; Shanghai, China) was obtained from AS-BI staining solution (B1) and FBB staining solution (B2) mixed at a ratio of 1:1. Then, cells were incubated in freshly prepared ALP staining solution in a humidified incubator for 20 min, away from light. The cells were washed with PBS again and counterstained with nuclear fast red staining solution for 1 min, followed by PBS rinsing. Cell staining was observed and photographed with a microscope (IX50, Olympus).

### Detection of the Nucleo-cytoplasmic Distribution of LINC01133

The DPSCs adherent to the disk wall were treated with 300 ul cell lysis buffer containing 10 ul RNase inhibitor (RI), and put in an ice bath for 10 min before centrifugation at 4°C and 500 g for 5 min. The supernatant was collected for another round of centrifugation for 10 min. After that, the supernatant comprised the cytoplasm and the sediments consisted of the cells’ nuclei. The experiment was conducted based on the Ambion PARISTM kit manufacturer’s instructions (Invitrogen; Carlsbad, CA, USA). The nuclei and cytoplasm of DPSCs were isolated, from which the RNA was extracted using qRT-PCR. The internal control for nuclei was U6 and that for cytoplasm was GAPDH. Each experiment was repeated 3 times.

### Cell Transfection

After 21 days of osteogenic induction, DPSCs were used for cell transfection. Overexpression or silencing of LINC01133 or AKT3 in DPSCs was achieved through transfection with 4 μg of pcDNA3.1-LINC01133, sh-LINC01133, pcDNA3.1-AKT3 and sh-AKT3 (Hanbio Biotechnology; Shanghai, China). The expression of miR-199b-5p was modified in DPSCs through transfection with 50 nmol of miR-199b-5p mimic or 100 nmol of miR-199b-5p inhibitor (Guangzhou RiboBio; Guangzhou, China). Following transfection, subsequent assays were conducted for 48 h. All transfection was carried out according to the Lipofectamine 2000 kit (Invitrogen) manufacturer’s instructions. The transfected cells were suspended in 10% FBS-supplemented DMEM, and seeded in 24-well plates (1 x 10^5^ cells/well) for incubation (5% CO_2_, humidity of 95%, 37°C).

### Groups

The cells were grouped as follows: blank group (untreated), pcDNA3.1 group (transfected with pcDNA3.1 vector), pcDNA3.1-LINC01133 group (transfected with pcDNA3.1-LINC01133), sh-NC group (transfected with the sh-NC plasmid), sh-LINC01133 group (transfected with sh-LINC01133), pcDNA3.1-AKT3 group (transfected with pcDNA3.1-AKT3), NC mimic group (transfected with NC mimic), miR-199b-5p mimic group (transfected with miR-199b-5p mimic), NC inhibitor group (transfected with NC inhibitor), miR-199b-5p inhibitor group (transfected with miR-199b-5p inhibitor), and miR-199b-5p mimic + pcDNA3.1-LINC01133 group (transfected with miR-199b-5p mimic and pcDNA3.1-LINC01133).

### qRT-PCR

DPSCs were lysed by TRIzol reagent for RNA extraction. RNA extract (5 μl) was diluted 20 times with RNase-free ultrapure water, and the optical density of the samples at 260 nm/280 nm in ultraviolet spectrophotometry was read to determine the concentration and purity of RNA. An OD260/OD280 ratio of 1.7 to 2.1 indicated that the isolated RNA was qualified for the following experiments. A cDNA template was synthesized by reverse transcription, and real-time quantitative RT-PCR assay was performed with ABI 7500 real-time PCR system based on the following conditions: pre-denaturation at 95°C for 10 min, followed by 40 cycles of denaturation at 95°C for 10 s, annealing at 60°C for 20 s and extension at 72°C for 34 s. Data were obtained after three independent experiments and analysed using the 2-ΔΔCt method. The formula is set as follows: ΔΔCt = [Ct (target gene) – Ct (reference gene)]1 experimental group – [Ct (target gene) – Ct (reference gene)] control group. All primers are listed in Table 1.

**Table 1:** Primer sequence for qRT-PCR to determine the expression levels of LINC01133, miR-199b-5p, AKT3, DMP1, DSPP, RUNX2, ALP, OSX, U6 and GAPDH.

Name of primer	Sequences
LINC01133-F	GTGTGTCCCTTGGTGGAGAG
LINC01133-R	TCCCAGATACCAGCGAAGGA
miR-199b-5p-F	CGCGCCCAGTGTTTAGACTAC
miR-199b-5p-R	AGTGCAGGGTCCGAGGTATT
RNUX2-F	TGGCTCAGTTCAGCAG
RNUX2-R	GTGCAGGGTCCGAGGT
ALP-F	CAGACGTTCCATACCCCCAC
ALP-R	GACCTTTGGCTCTCGACCAG
OSX-F	CTCAGGCCACCCGTTG
OSX-R	CATAGTGAACTTCCTCCTCAAGC
AKT3-F	ACCGCACACGTTTCTATGGT
AKT3-R	CCCTCCACCAAGGCGTTTAT
DMP1-F	TGAGTGAGTCCAGGGGAGATAA
DMP1-R	TTTTGAGTGGGAGAGTGTGTGC
DSPP-F	TTAAATGCCAGTGGAACCAT
DSPP-R	ATTCCCTTCTCCCTTGTGAC
U6-F	CTCGCTTCGGCAGCACATATACT
U6-R	ACGCTTCACGAATTTGCGTGTC
GAPDH-F	TGCACCACCAACTGCTTAG
GAPDH-R	GGATGCAGGGATGATGTTC

F: forward; R: reverse.

### Western Blot

DPSCs cells were washed 3 times with pre-cooled PBS buffer. After that, the cells were dissolved with protein extraction lysis buffer in a 100 μl/50 ml culture flask on ice for 30 min. After centrifugation for 10 min at 12,000 rpm and 4°C, the supernatant was collected, aliquoted, and stored in centrifuge tubes (0.5 ml) at -20°C. Bovine serum albumin (BSA, 2 μg/μl) was diluted into 20 μg/μl, 15 μg/μl, 10 μg/μl, 5 μg/μl, 2.5 μg/μl or 0 μg/μl with PBS. A bicinchoninic acid protein assay kit (BCA, Beyotime Biotechnology) was used for determining the protein concentration. In brief, BCA reagent A and B were mixed at a ratio of 50:1. A total of 2 μl of protein samples were diluted with 18 μl of double-distilled water, with 2 replicate wells. Then 200 μl mixture of BCA reagent A and B was mixed with 10 μl diluted standard and 10 μl protein sample in each well of the 96-well plate, and the plate was shaken gently and incubated for 30 min at 37°C. After the incubation, the plate was cooled down to room temperature. The absorbance was measured at 495 nm using a microplate reader, and the protein concentration was calculated based on a standard curve.

Electrophoresis was then run at 60 V and changed to 120 V after bromophenol blue entered the separation gel (1 to 2 h, 4°C). Subsequently, the proteins were transferred from gel to a polyvinylidene fluoride (PVDF) membrane using the wet-transfer method and electrotransferred for 2 h at 4°C. Then the PVDF membrane was sealed with 5% nonfat milk in TBST buffer for 1 to 2 h. The membranes were incubated with the primary antibodies of rabbit anti-human runt-related transcription factor 2 (RUNX2, #12556, 1:1000), AKT3 (#14982, 1:1000, Cell Signaling; Boston, MA, USA), ALP (ab133602, 1:10000), osterix (OSX, ab209484, 1:1000, Abcam, Cambridge, MA, USA), DSPP rabbit anti human (ab272929, 1:500, Abcam) and DMP1 rabbit anti human (DF8825, 1:1000; Affinity Biosciences; Jiangsu, China) at 4°C overnight followed by TBST washing (3 x 10 min). Afterwards, the cells were treated with goat anti-rabbit IgG (#7074, 1:1000, Cell Signaling Technology) for 1 h at room temperature. Chemiluminescent assay was performed to analyze protein bands, followed by data analysis. GAPDH (#5174, 1:1000, Cell Signaling Technology) was used as the internal reference.

### Luciferase Reporter Gene Assay

The target sites for binding of LINC01133 and miR-199b-5p or of miR-199b-5p and AKT3 were analyzed using online software starBase (http://starbase.sysu.edu.cn/). According to the prediction, wild-type (WT) and mutant-type (MT) sequences of the binding sites of LINC01133 and miR-199b-5p or of miR-199b-5p and AKT3 were designed, cloned into pGL3-Promoter vector, and designated as LINC01133-WT, LINC01133-MUT, AKT3-MT, and AKT3-MUT, respectively. Then, the vectors were separately co-transfected with miR-199b-5p mimic into 293T cells using Lipo293 (Beyotime Biotechnology). About 48 h later, luciferase activity was evaluated using a luciferase kit (Amyjet Scientific; Wuhan, China).

### Statistical Analysis

Differences between groups were estimated by the t-test, whereas multiple comparisons were performed by using one-way ANOVA, followed by Tukey’s multiple comparisons test. SPSS17.0 and GraphPad Prism 5.0 were used for data analysis. Statistical significance was set at p < 0.05.

## Results

### LINC01133 decreased, but miR-199b-5p increased during the OD of DPSCs

**Fig 1:**
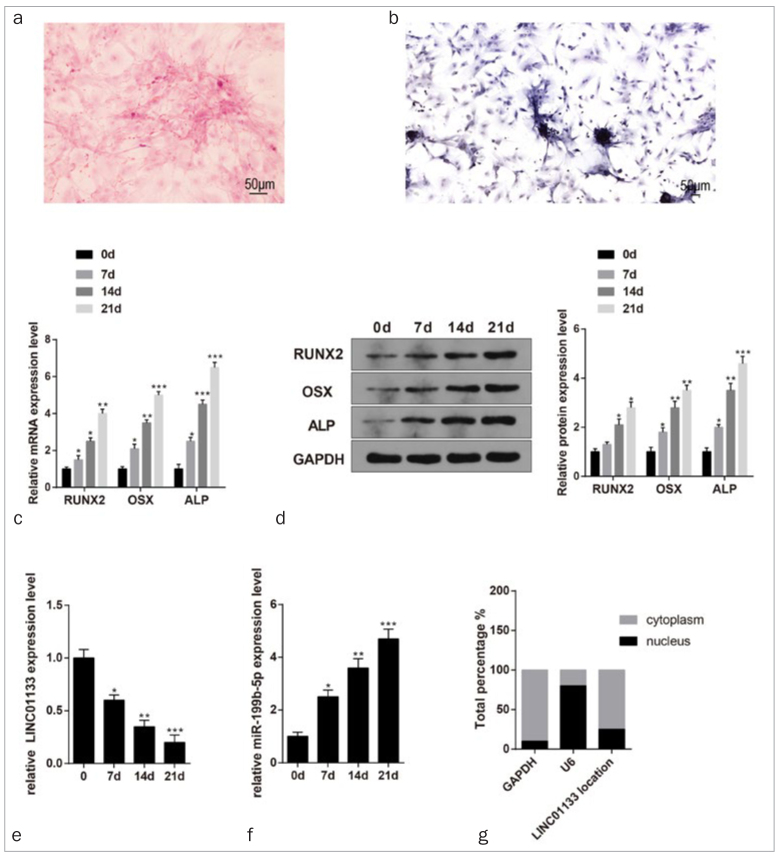
The expression patterns of LINC01133 and miR-199b-5p in the OD of DPSCs. NoteAfter 21 days of OD, the results of ARS and ALP staining were evaluated (a–b). qRT-PCR and Western blotting were applied to determine the levels of osteogenic markers (RUNX2, OSX and ALP) (c–d). The mRNA expressions of LINC01133 and miR-199b-5p were inspected by qRT-PCR (e–f). Nucleo-cytoplasmic distribution of LINC01133 in DPSCs (g). *p < 0.05, **p < 0.01, ***p < 0.001 vs 0 days; OD, osteogenic differentiation; DPSCs, dental pulp stem cells; ARS, alizarin red S; ALP, alkaline phosphatase; RUNX2, runt-related transcription factor 2; OSX, osterix. Measurement data were expressed as mean ± SD. Comparisons among multiple groups were analyzed using one-way ANOVA and Tukey’s post-hoc test. Experiments were repeated 3 times.

The isolated DPSCs were stained with ARS and ALP staining after 21 days of osteogenic induction. The extracted DPSCs were stained by ARS and ALP to assess the mineralized nodules (Figs 1a and 1b). Further analyses of qRT-PCR and Western blot showed increased expressions of osteogenic marker genes (RUNX2, OSX and ALP) during the differentiation process (Figs 1c and 1d, p < 0.05). In addition, qRT-PCR showed that LINC01133 dramatically decreased (Fig 1e, p < 0.05), while miR-199b-5p expression increased (Fig 1f, p < 0.05) during the differentiation process of DPSCs. Detection of the nucleo-cytoplasmic distribution of LINC01133 showed that LINC01133 was mainly distributed in the cytoplasm of DPSCs (Fig 1g).

### LINC01133 overexpression suppressed the OD of DPSCs

**Fig 2:**
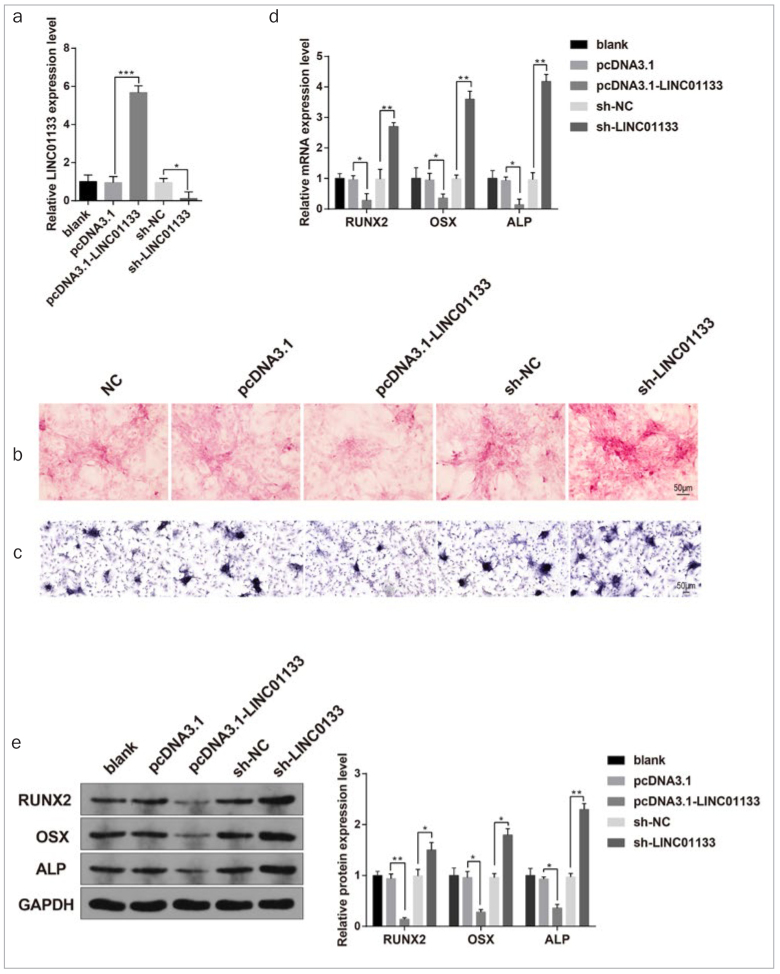
The effects of LINC01133 on the OD of DPSCs. qRT-PCR was used to detect LINC01133 expression in DPSCs (a). ARS staining and ALP staining for DPSCs (b–c). The levels of osteogenic markers (RUNX2, OSX and ALP) were determined by qRT-PCR and Western blot (d–e). *p < 0.05, **p < 0.01, ***p < 0.001; OD, osteogenic differentiation; DPSCs, dental pulp stem cells; ARS, alizarin red S; OSX, osterix; ALP, alkaline phosphatase; RUNX2, runt-related transcription factor 2. Measurement data were expressed as mean ± SD. Comparisons among multiple groups were analyzed using one-way ANOVA and Tukey’s post-hoc test. Experiments were repeated 3 times.

After induction of OD for 21 days, pcDNA3.1-LINC01133 and sh-LINC0113 were introduced into the DPSCs, after which the transfection efficiency of LINC01133 expression was quantified by qRT-PCR. LINC01133 expression increased markedly in the pcDNA3.1-LINC01133 group, while LINC01133 expression declined in the sh-LINC01133 group, compared with the pcDNA3.1 group or sh-NC group (Fig 2a, p < 0.05). Moreover, ARS staining and ALP staining showed that silencing of LINC01133 led to an increased number of mineralised nodules and overexpression of LINC01133 resulted in decreased mineralised nodules (Figs 2b and 2c). In contrast, increased LINC01133 expression substantially inhibited the OD of DPSCs (Figs 2b and 2c). Further, qRT-PCR and Western blot analyses confirmed that DPSCs with overexpression of LINC01133 showed dramatically decreased RUNX2, OSX and ALP, while silencing of LINC01133 in DPSCs led to increased expression of these osteogenic markers (Figs 2d and 2e, p < 0.05).

### LINC01133 competed with AKT3 to bind miR-199b-5p

**Fig 3:**
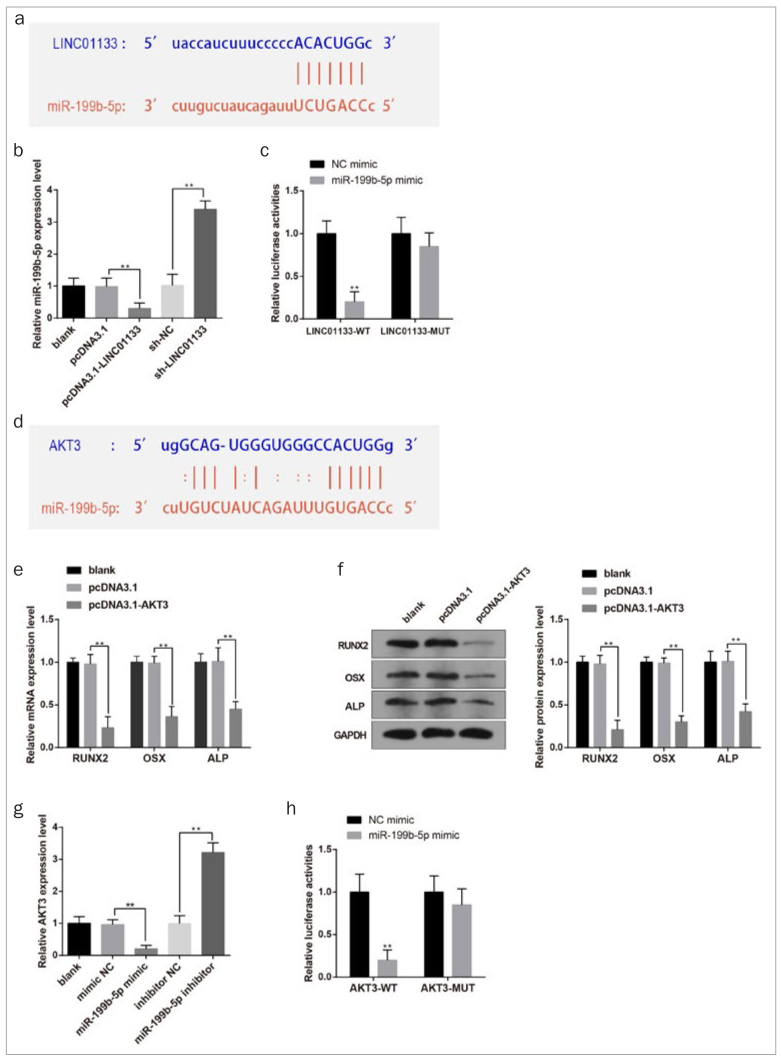
The target relationship among LINC01133, miR-199b-5p and AKT3. Online software starBase predicted the binding sites of LINC01133 and miR-199b-5p (a). The effects of LINC01133 overexpression and deficiency on miR-199b-5p expression were measured (b). The target relationship between LINC01133 and miR-199b-5p was verified by luciferase reporter gene assay (c). Potential binding sites between miR-199b-5p and AKT3 were predicted by starBase (d). The mRNA and protein levels of osteogenic markers were detected after AKT3 was overexpressed (e–f). Detection of the relative AKT3 expression (g). The target relationship between miR-199b-5p and AKT3 was measured by luciferase reporter gene assay (h). *p < 0.05, **p < 0.01. Measurement data were expressed as mean ± SD. Comparisons among multiple groups were analyzed using one-way ANOVA and Tukey’s post-hoc test. Experiments were repeated 3 times.

The online software starBase predicted the potential binding sites of miR-199b-5p and LINC01133 (Fig 3a). Moreover, the gain- and loss-of-function experiments of LINC01133 showed that LINC01133 negatively regulated miR-199b-5p expression (Fig 3b). Luciferase reporter gene assay demonstrated that cells in the LINC01133-MUT + miR-199b-5p mimic group showed unchanged luciferase activity compared with the LINC01133-MUT + NC mimic group. However, the luciferase activity of cells transfected with LINC01133-WT was drastically suppressed after transfection of miR-199b-5p mimic (Fig 3c, p < 0.01, vs. the LINC01133-WT + NC mimic group). These data demonstrated the target relationship between LINC01133 and miR-199b-5p.

In addition, starBase clarified the potential binding sites between miR-199b-5p and AKT3 (Fig 3d). As depicted in Figs 3e and 3f, elevation of AKT3 expression resulted in inhibited mRNA and protein levels of osteogenic marker genes, indicating the inhibitory role of AKT3 on OD of DPSCs (p < 0.01). To further investigate the relationship between miR-199b-5p and AKT3, overexpression or silencing of miR-199b-5p was achieved in DPSCs. As shown in Fig 3g, overexpression of miR-199b-5p dramatically reduced AKT3 expression (p < 0.01). The target relationship between miR-199b-5p and AKT3 was identified by luciferase reporter gene assay. Results denoted that overexpression of miR-199b-5p effectively downregulated the luciferase activity of cell transfected with AKT3-WT reporter (p < 0.01), but could not affect the luciferase activity of cells transfected with AKT3-MUT (Fig 3h). These data suggested that LINC01133 sponges miR-199b-5p, and miR-199b-5p could directly regulate AKT3.

### LINC01133 restricted OD of DPSCs through a ceRNA network

**Fig 4:**
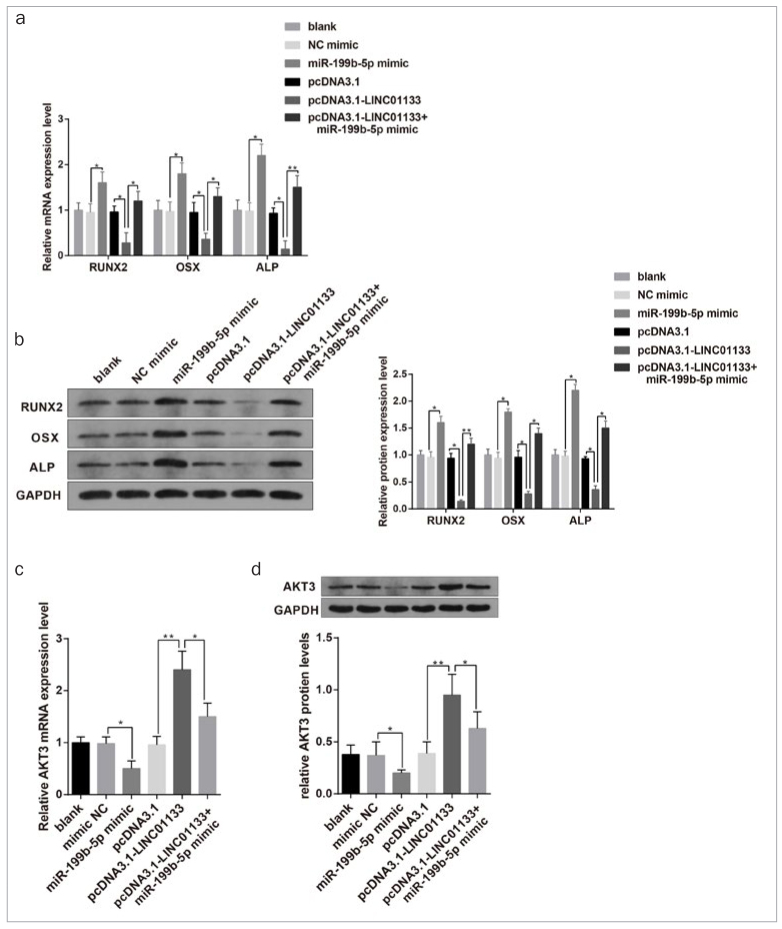
LINC01133/miR-199b-5p/AKT3 inhibits the OD of DPSCs. After transfection with pcDNA3.1-LINC01133 or/and miR-199b-5p mimic, the mRNA and protein levels of osteogenic markers (RUNX2, OSX and ALP) were measured (a–b). The mRNA and protein levels of AKT3 were assessed (c–d). *p < 0.05, **p < 0.01. OD, osteogenic differentiation; DPSCs, dental pulp stem cells; RUNX2, runt-related transcription factor 2; OSX, osterix; ALP, alkaline phosphatase. Measurement data were expressed as mean ± SD. Comparisons among multiple groups were analyzed using one-way ANOVA and Tukey’s post-hoc test. Experiments were repeated 3 times.

To further ascertain the modulatory mechanism of LINC01133-regulated OD of DPSCs, rescue analysis was performed to explore the interactions among LINC01133, miR-199b-5p and AKT3 during the OD of DPSCs. Results revealed that miR-199b-5p overexpression greatly augmented the level of osteogenic markers in DPSCs, while LINC01133 overexpression significantly restrained the expressions of these genes in DPSCs. However, introduction of pcDNA3.1-LINC01133 and miR-199b-5p mimic synergically increased the expression of osteogenic markers in DPSCs compared with transfection with pcDNA3.1-LINC01133 alone, suggesting miR-199b-5p could reverse the inhibition of LINC01133 overexpression on the OD of DPSCs (Figs 4a and 4b, p < 0.05). qRT-PCR and Western Blot demonstrated that the effect of LINC01133 and miR-199b-5p on odontogenic markers DSPP and DMP1. The results showed that DPSCs transfected with pcDNA3.1-LINC01133 had decreased expressions of DSPP and DMP1, while cells transfected with miR-199b-5p mimic had elevated expressions of DSPP and DMP1. In addition, co-transfection of pcDNA3.1-LINC01133 with miR-199b-5p mimic led to elevated expression of DSPP and DMP1 compared with cells transfected with pcDNA3.1-LINC01133 alone (Supplemented figure 1A-B).

Meanwhile, the levels of AKT3 were repressed in miR-199b-5p-overexpressed DPSCs, but increased distinctly in LINC01133-overexpressed DPSCs. However, co-transfection of pcDNA3.1-LINC01133 and miR-199b-5p mimic decreased AKT3 mRNA and protein levels, compared to pcDNA3.1-LINC01133 transfection alone (Figs 4c and 4d, p < 0.05). These results indicated that miR-199b-5p could eliminate LINC01133-mediated increases of AKT3 expression. Taken together, LINC01133 regulated the OD of DPSCs through upregulating AKT3 expression by absorbing miR-199b-5p.

## Discussion

By serving as precursor cells of odontoblasts, DPSCs have close relationship with dentin differentiation and mineralised tissue formation,^21,22^ indicating DPSCs are potentially involved in dentin regeneration. In this regard, it is relevant to explore the regulation of OD of DPSCs. Thus, DPSCs are extracted from healthy dental pulp to assess their OD ability, with the aim of exploring possible regulatory factors that may be involved in the OD of DPSCs. The data presented here showed that LINC01133 functions as an endogenous sponge of miR-199b-5p to regulate AKT3 expression, thus decreasing the OD of DPSCs.

LncRNAs are widely recognised as key regulators of multiple cellular biological processes, including cell differentiation.^9,35^ LncRNA H19 was demonstrated to be an important regulatory factor in odontogenic differentiation of human DPSCs via regulation of S-adenosylhomocysteine hydrolase.^38^ LncRNA ANCR was shown to modulate DPSC osteogenic, neurogenic and adipogenic differentiation.^14^ Additionally, LncRNA FAM96B was reported to attenuate DPSC senescence and aggravate osteogenic proliferation and differentiation.^20^ Importantly, previous studies corroborated that LINC01133 is involved in cell proliferation, apoptosis, cell cycle, and colony formation.^12,43^ Moreover, a previous study reported the promotive effect of LINC01133 on the OD of adipose-derived MSCs,^29^ but its role in OD of DPSCs remains to be elucidated. The gain- and loss-of-function study of LINC01133 in this study demonstrated that overexpression of LINC01133 impeded OD, implicating the role of LINC01133 in the pathogenesis of DPSCs. Further analysis discovered that LINC01133 expression was negatively related to the expression of the osteogenic marker genes RUNX2, OSX, ALP, DSPP and DMP1. These results indicate that LINC01133 is implicated in DPSC osteogenesis.

LncRNAs can act as ceRNAs to play significant roles in regulating mRNA expression by competing for shared miRNAs.^37^ For instance, a study by Jia et al^13^ showed that LINC00707 acted as a ceRNA to facilitate osteogenesis of human bone marrow-derived MSCs via sponge miR-370-3p. More importantly, Zhong et al^44^ demonstrated that LncRNA CCAT1 sponged miRNA-218 to accelerate human DPSCs proliferation and differentiation. Furthermore, LncRNAs (AK039312 and AK079370) have been reported to suppress osteoblast differentiation via miR-199b-5p.^36^ The prediction of the online software starBase and functional analyses in this study proved miR-199b-5p to be the downstream target of LINC01133 in DPSCs. Currently, miR-199b-5p was known to accelerate cell proliferation by targeting DDR1 in breast cancer.^33^ Previously, miR-199b-5p hindered OD in ligamentum flavum cells by targeting JAG1.^28^ As opposed to a previous study,^28^ ours found that miR-199b-5p overexpression elevated the expression of osteogenic markers. In agreement with our observation, Zhao et al^42^ showed that miR-199b-5p can promote osteogenesis in human bone marrow stromal cells through the GSK-3β/β-catenin signaling pathway, which consistently highlighted the role of miR-199b-5p in regulating osteogenesis. This is the first study to determine miR-199b-5p as a key regulator in the OD of DPSCs, but the downstream target genes regulating the differentiation process remain largely unknown. AKT3 is a homologous gene that belongs to the serine/threonine protein kinase AKT subfamily.^32^ A previous study^3^ showed that LY3023414 could inhibit the downstream genes associated with osteogenesis, including AKT1/2. In this research, we further revealed that AKT3 hindered the OD of DPSCs. Also, AKT3 was a downstream target of miR-199b-5p in modulating DPSC osteogenesis as identified by bioinformatics and luciferase analysis. Thus, miR-199b-5p serves as a potential target of LINC01133, but also an upstream target of AKT3. Subsequently, the interactions among LINC01133, miR-199b-5p and AKT3 were demonstrated by rescue experiments. The results showed that miR-199b-5p could reverse the inhibitory effects of LINC01133 on regulating OD of DPSCs. Furthermore, LINC01133 sponged miR-199b-5p to regulate AKT3 expression, as miR-199b-5p overexpression decreased AKT3 expression in LINC01133-expressed DPSCs. Collectively, LINC01133 promoted OD of DPSCs through upregulating AKT3 expression via competitively binding miR-199b-5p.

## Conclusion

Our study provides novel insights into the osteogenesis mechanisms of DPSCs. It is the first study to illustrate that LINC01133 functions as an inhibitor of osteoblast differentiation via positive regulation on AKT3 expression by sponging miR-199b-5p. Here, the LINC01133/miR-199b-5p/AKT3 axis might provide novel diagnostic biomarkers and new clues for therapeutic strategies for dentin regeneration in the dental pulp.
